# The mental and emotional status after radical cystectomy and different urinary diversion orthotopic bladder substitution versus external urinary diversion after radical cystectomy: A propensity score‐matched study

**DOI:** 10.1111/iju.15586

**Published:** 2024-09-24

**Authors:** Giuseppe Palermo, Francesco Pio Bizzarri, Eros Scarciglia, Emilio Sacco, Koosha Moosavi Seyed, Pierluigi Russo, Filippo Gavi, Battista Filomena Giovanni, Francesco Rossi, Marco Campetella, Angelo Totaro, Nazario Foschi, Marco Racioppi

**Affiliations:** ^1^ Department of Urology Fondazione Policlinico Universitario Agostino Gemelli IRCCS Rome Italy; ^2^ Catholic University of Sacred Heart Rome Italy; ^3^ Department of Urology Ospedale Isola Tiberina—Gemelli Isola Rome Italy; ^4^ Department of Medicine and Translational Surgery Università Cattolica del Sacro Cuore Rome Italy

**Keywords:** bladder cancer, orthotopic neobladder, quality of life, urinary diversion

## Abstract

**Objectives:**

The quality of life in patients undergoing radical cystectomy and urinary diversion is gaining importance. Nowadays a broad consensus about the best urinary diversion does not exist. This study presents an all‐round analysis of the impact of two types of urinary diversion on life's psycho‐social aspects in patients undergoing radical cystectomy.

**Methods:**

This is an observational, single‐centre, prospective study. Eligible participants underwent radical cystectomy and urinary diversion for bladder cancer in our department from January 2020 and February 2024. Of 130 included patients, 90 (45 with Bricker's ureteroileocutaneostomy and 45 received orthotopic bladder replacement) patients were matched and the study groups were well balanced for the baseline‐matched variables. Patients completed 4 questionnaires (EORTC QLQ‐C30, PGWBI, HADS, PSQI) at three different times: before the surgical procedure, and at 3 and 12 months.

**Results:**

Time shows a statistically significant effect (*p* < 0.0005) on four of the five functional scales explored (Physical Functioning, Role Functioning, Emotional Functioning, Social Functioning), and for all the nine symptoms/items' scales (*p* < 0.0005) and the Global Health Status (*p* = 0.003) in EORTC QLQ‐C30. Neobladder's group shows a statistically significant improvement on the scales of Physical Functioning, Role Functioning, and Social Functioning, and for symptoms of nausea (*p* = 0.0027), pain (*p* = 0.0005), dyspnea (*p* = 0.012), insomnia (*p* = 0.004), constipation (*p* = 0.003).

**Conclusion:**

We do not find a better urinary diversion in absolute terms. Neobladder obtained better results only for specific items and features. The urinary diversion's choice must be made in concert by the patient, the caregiver, and health professionals with adequate counseling.

**Abbreviations & Acronyms**
HADS: Hospital Anxiety and Depression Scale
PGWBI: Psychological General Well‐Being Index
PSQI: Pittsburg Sleep Quality Index

## INTRODUCTION

Bladder cancer is the seventh most diagnosed cancer in the male population worldwide and one of the most expensive cancers in average healthcare costs.^1^ Treatment choice could be influenced by many aspects, on which healthcare professionals have to counsel with both the patient and the caregiver.^2^, ^3^ The role of quality of life in patients undergoing radical cystectomy and urinary diversion is gaining importance.^4^ There are few studies and considerations about it, and even less regarding the mental and the emotional health components that trouble these patients. In addition to a personal history of bladder cancer, they have a new way of fulfilling physiological needs, particularly those regarding urination.^5^ About urinary diversion, no one is universally recognized as superior, whereas the treatment of choice for muscle‐invasive bladder cancer remains radical cystectomy, even in the case of the elderly.^6^ About treatment and follow‐up of bladder cancer, the literature is established and rich.^7^ We can choose from many urinary diversions to ensure easier and better management, QoL‐based on, too.^8^ And trying to fulfill patients' physical, social, and psychological needs, in addition to the best disease control. However, many aspects of daily life are not considered.^9^ Such as sexual activity, which is compromised in equal measure for the continent and noncontinent diversions.^10^ Furthermore, the quality of life in bladder cancer is worse compared to other pelvic cancers.^11^ Despite this, literature is poor in studies on this topic and evidence is low quality and with short‐term follow‐up.^12^ The main objective of this study is to describe the mental and emotional health status of patients undergoing radical cystectomy and urinary diversion for bladder cancer, comparing the impact on the Quality of Life (ansia, depression, and quality of sleep) of Orthotopic Bladder substitution versus External urinary diversion.

## METHODS

### Study design and patients

This is an observational, single‐centre, prospective study, performed in at university reference hospital, comparing the impact on the Quality of Life of Orthotopic Bladder substitution versus External urinary diversion. Eligible participants (aged ≥18 years) underwent radical cystectomy for bladder cancer and urinary diversion. The exclusion criteria were the choice of a different urinary diversion, such as ureterocutaneostomy; the inability to understand the tests with multiple cross‐response; the noncompliance with the clinical and instrumental follow‐up. Institutional review board approval has been obtained. The study has been approved by the Ethics Committee (ID 2882, approved by the EC on 05/12/2019).

### Procedures

A total of 130 patients, 64% males and 36% females, have been included in the study. Bricker's diversion has been performed on 70 (53.8%) patients and “VIP” on 60 (46.2%).

We selected for “VIP”, patients under 75 years of age, with no history of previous major abdominal surgery, with no carcinoma in situ in the urethra and ureteral sections and with normal renal function. Radical cystectomies (including locoregional lymphadenectomy) for bladder cancer and urinary diversion were performed in 40 (31%) patients with robotic approach but all reconstructive precedures were performed with open approach. According to previous demonstrations, we assume that the “VIP” is a safe option with comparable outcomes to other bladder orthotopic reconstruction.^13^ In our department we performed a “VIP” urinary diversion for Bladder cancer, because we have a tradition with this type of diversion from decades. The choice between the two diversions was purely clinical. The two diversions were built with the extracorporeal method, regardless of whether cystectomy was robotic or open method, because previous studies already showed their overlap.^14^ All patients received 4 questionnaires and were asked to complete them at baseline (days before surgery), and postoperatively at months 3 and 12 from surgery. They completed them in a self‐administrated filling way, in which the examining physician was available for patients in case they needed further information. The questionnaires were: the European Organization for Research and Treatment of Cancer quality of life questionnaire (EORTC QLQ‐C30), the Psychological General Well‐Being Index (PGWBI), the Hospital Anxiety and Depression Scale (HADS) and Pittsburg Sleep Quality Index (PSQI).

### Surgical description

The VIP neobladder procedure is primarily based on its original description, with minor surgical adjustments. Essentially, a 40–50 cm section of the lower ileum, located about 15–20 cm from the junction of the small and large intestines, is shaped into a sideways “U.” The bowel is then opened lengthwise along the side closest to its blood supply, rather than the opposite side. A short, tubular section (about 3–4 cm) is created from the lower end of the bowel to form the bladder outlet. The upper part of the bowel is rolled into a spiral and stitched together to create the main pouch. Both ureters are then connected to the top of the pouch. The pouch is tested during surgery, typically holding 200–400 mL. Temporary tubes are placed in the ureters (catheters of Bracci 6 Ch or 8Ch) and urethra (Catheter like Dufour 24CH). The Bracci's catether were removed 14 days after surgery and Dufour was removed 30 days after surgery.^13^

### Questionnaires

All patients received 4 questionnaires and were asked to complete them at baseline (days before surgery), and postoperatively at months 3 and 12 from surgery. They completed them in a self‐administrated filling way, in which the examining physician was available for patients in case they needed further information. The questionnaires were: the European Organization for Research and Treatment of Cancer quality of life questionnaire (EORTC QLQ‐C30), the PGWBI, the HADS and the PSQI.

#### EORTC QLQ‐C30

The EORTC QLQ‐C30 consists of five functional scales (physical, emotional, cognitive, social, and role functioning), three symptom scales (fatigue, pain, and nausea/vomiting), a global health status/QoL scale, and six single items (dyspnea, insomnia, appetite, constipation, diarrhea). All the scales range in score from 0 to 100. A high scale score represents a higher response level. In more detail: a high score for a functional scale represents a healthy level of functioning, and a high score for the global health status represents a high QoL. On the other side, a high score on a symptom scale represents a high level of symptomatology.^15^

#### The PGWBI

The PGWBI is a measure of the level of subjective psychological well‐being. In detail, it assesses self‐representations of intrapersonal affective or emotional states reflecting a sense of subjective well‐being or distress and thus captures what we could call a subjective perception of well‐being. Consisting of 22 standardized items, the tool produces a single measure of the psychological well‐being. The full measure also provides subscales to assess the following domains: anxiety, depression, positive well‐being, self‐control, general health, and vitality. Higher scores indicate greater psychological well‐being.^16^

#### Hospital Anxiety and Depression Scale

The HADS is a 14‐item measure designed to assess anxiety and depression symptoms in medical patients, with an emphasis on reducing the impact of physical illness on the total score. The depression items tend to focus on the anhedonic symptoms of depression. Items are rated on a 4‐point severity scale. The HADS produces two scales, one for anxiety (HADS–A) and one for depression (HADS–D), differentiating the two states. Scoring for each item ranges from 0 to 3, with 3 denoting the highest anxiety or depression level. A total subscale score of >8 points out of a possible 21 denotes considerable symptoms of anxiety or depression.^17^

#### The PSQI

The study involved participants filling out a questionnaire PSQI.

This PSQI test, designed by Buysse et al., is a self‐assessment tool where people report on their sleep quality over the past month.^18^ The PSQI provides a total score and scores for seven different sleep aspects. These aspects include how good the person feels their sleep is, how long it takes them to fall asleep, total sleep time, sleep efficiency, sleep disturbances, use of sleep medication, and daytime dysfunction. Each aspect is scored between 0 and 3, with a higher total score (ranging from 0 to 21) indicating worse sleep quality. Research has shown that a PSQI score above 5 is a good indicator of poor sleep across various populations, including older adults.^19^

### Statistical analyses

The sample has been described in its clinical and demographic characteristics by applying descriptive statistics techniques. In order to compare and create a similar group between patients a Propensity Score matching was conducted. By matching 90 patients with similar propensity scores, PSM helps to minimize bias when comparing outcomes between patients with Neobladder and patients with ileal conduit. The passage describes how the researchers used logistic regression to calculate propensity scores based on patient characteristics. They also checked to ensure the characteristics and propensity scores were well balanced between the matched groups. Qualitative variables have been described with absolute frequencies and percentages. Quantitative variables have been summarized with mean and standard deviation. Comparisons between the two diversions groups have been performed by applying the Chi‐square test (or the Fisher's exact test) for categorical variables. For continuous, not normally distributed, variables, the Mann–Whitney test has been applied. Comparisons of QoL questionnaires results have been performed applying the two‐way mixed ANOVA, where the between‐subject factor was the type of urinary diversion and the within‐subject factor was the time of evaluation (our 3 times were: *T*_0_ = baseline, *T*_1_ = 3 months, and *T*_2_ = 12 months after the surgery). A *p*‐value < 0.05 was considered statistically significant. Analyses were performed using SPSS ver. 25.

## RESULTS

### Matching procedure and baseline characteristics

**FIGURE 1:**
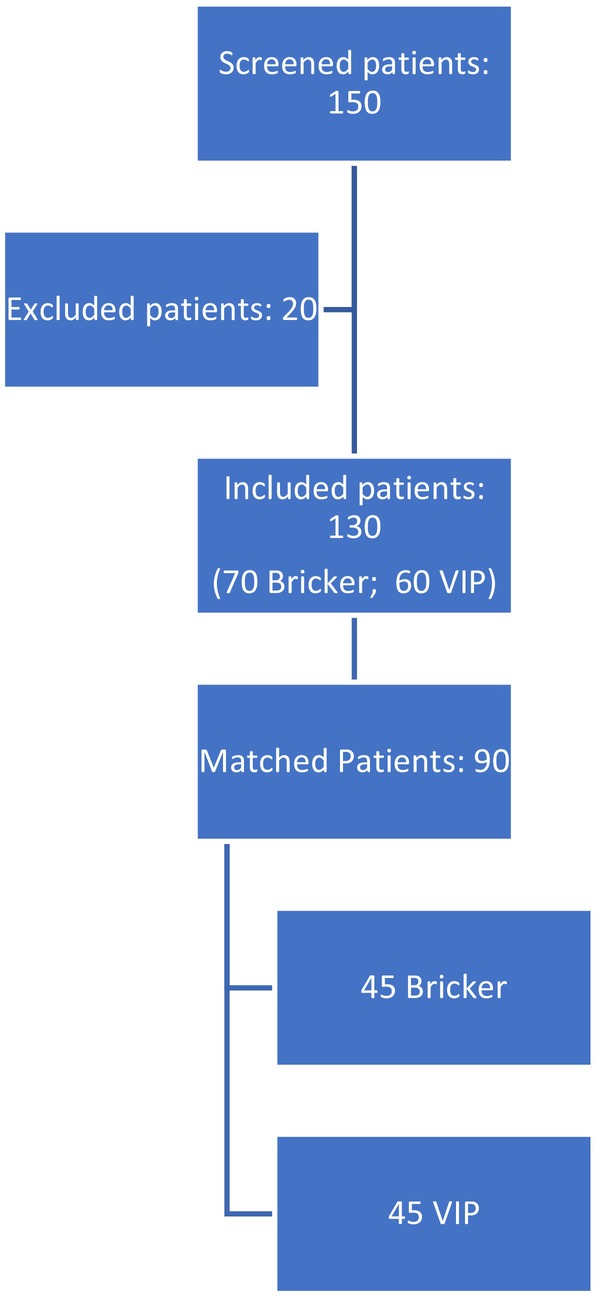
Study flowchart.

Out of 150 screened patients, 130 remained eligible after patients selection and 90 patients were matched according to Propensity score (Figure 1). Patients with ileal conduit group showed a lower median age (75 vs. 63), an higher Charlson Comorbidity Index (6.31 ± 1.6 vs. 4.8 ± 1.32) and a lower rate of preoperative chemotherapy (25% vs. 64.7%) than the “VIP” group.

After performing a propensity score matching a statistically significant difference between the two groups is the proportion of patients that have completed chemotherapy (*p*‐value: 0.006), the median Age (*p*‐value: 0.0043) and Charlson's comorbidity index (*p*‐value: <0.0005) (Table 1).

**TABLE 1:** Comparison of baseline characteristics between groups in the unmatched and matched populations.

	Unmatched population				Matched population			
	Total	Bricker [70; 53.8%]	VIP [60; 46.2%]	*p*‐value	Total	Bricker [45; 50%]	VIP [45; 50%]	*p*‐value
Sex (*n*; %)								
M	83 (64%)	80%	83%	0.59	53 (58.8%)	57%	64%	0.61
F	47 (36%)	20%	17%		47 (41.2%)	43%	46%	
Age (*n*; %)								
Mean ± SD	71.2 ± 9.7	74 ± 8.35	62.6 ± 8.00	**<0.0005**	73 ± 8.7	76 ± 6.3	70 ± 7.6	**0.0043**
Median	72	75	63		74	78	71	
Charlson comorbidity index								
Mean ± SD	5.43 ± 1.6	6.31 ± 1.6	4.8 ± 1.32	**<0.0005**	5.1 ± 2	5.3 ± 1.7	4.2 ± 1.24	**<0.0005**
Median	6	6	5		5	5	4	
Histological (*n*; %)								
Cis	15 (11.5%)	5%	10.8%	0.09^a^	2 (2.2%)	1 (2.2%)	3 (6.6%)	0.076
Negative	17 (13.1%)	15%	2%		0 (0.0%)	0 (0.0%)	0 (0.0%)	
Poorly diff	9 (6.9%)	6.2%	0.0%		0 (0.0%)	0 (0.0%)	0 (0.0%)	
Squamous	5 (3.8%)	3%	0.0%		4 (4.4%)	3 (6.6%)	0 (0.0%)	
Urothelial	70 (53.8%)	61.2%	70.6%		80 (88.9%)	30 (66.7%)	34 (75.5%)	
Urothelial + Cis	14 (14%)	9.6%	16.6%		4 (4.4%)	10 (22.2%)	8 (17.9)	
*T* (*n*; %)								
Ta (HG)	2 (1.5%)	1.8%	0%	0.880	0 (0.0%)	0 (0.0%)	0 (0.0%)	0.670
1	25 (19.2%)	24.6%	36.3%		14 (15.5%)	4 (8.8%)	3 (6.6%)	
2	70 (53.8%)	41%	45.6%		60 (66.6%)	35 (77.7%)	37 (82.4%)	
3	18 (13.8%)	20%	12.5%		10 (11.1)	4 (8.8%)	3 (6.6%)	
4	10 (7.6%)	4.9%	0.0%		0 (0.0%)	0 (0.0%)	0 (0.0%)	
Cis	5 (3.8%)	7.7%	5.6%		6 (5.5%)	2 (4.4%)	2 (4.4%)	
*N* (*n*; %)								
0	86 (66.1%)	86.9%	87.5%	0.429^a^	74 (82.2)	36 (80%)	32 (71.1%)	0.39
1	38 (29%)	6.6%	6.3%		16 (17.6%)	9 (20%)	13 (28.9%)	
2	3 (2.3%)	1.6%	6.3%		0 (0.0%)	0 (0.0%)	0 (0.0%)	
3	3 (2.3%)	4.9%	0.0%		0 (0.0%)	0 (0.0%)	0 (0.0%)	
Chemotherapy								
No	89 (68.4%)	75%	35.3%	0.009	61 (67.7%)	38 (84.4%)	34 (75.6%)	0.006
Yes	41 (31.5%)	25%	64.7%		29 (23.3%)	7 (15.6%)	11 (24.4%)	
Relapse								
No	110 (84.6%)	86.1%	88.2%	0.333	78 (86.6%)	39 (86.6%)	41 (91.1%)	0.42
Yes	20 (15.38)	13.9%	11.8%		12 (13.4%)	6 (13.4%)	4 (8.9%)	
Clavien‐Dindo at 3 months								
1	80 (61.5%)	62%	50.6%	0.09	64 (71.1%)	33 (73.3%)	35 (77.7%)	0.078
2	10 (7.69%)	7.80%	12.4%		10 (11.1%)	4 (8.8%)	3 (6.6%)	
3a	20 (15.3%)	15%	30.3%		5 (5.6%)	3 (6.6%)	3 (6.6%)	
3b	23 (17.7%)	15.2%	4.7%		11 (12.2%)	5 (11.3%)	4 (9.1%)	
4a	7 (5.3%)	0.00%	2%		0 (0.0%)	0 (0.0%)	0 (0.0%)	
Clavien‐Dindo at 12 months								
1	84 (64.6%)	65.2%	58.2%	0.265	74 (82.2%)	38 (84.4%)	40 (88.8%)	0.42
2	25 (19.2%)	12.8%	16%		16 (17.8%)	7 (16.6%)	5 (11.2%)	
3a	16 (12.3%)	15.6%	17%		0 (0.0%)	0 (0.0%)	0 (0.0%)	
3b	5 (3.8%)	6.4%	8.8%		0 (0.0%)	0 (0.0%)	0 (0.0%)	

^a^ Fisher's exact test.

THe bold values identify the statically significant ones.

#### EORTC QLQ‐C30

The two‐way mixed ANOVA did not highlight any interaction between time and groups. Four of the five functional scales (Physical, Role, Emotional, and Social Functioning) show a higher score in *T*_2_ than in *T*_0_ for both groups, and the effect of time was statistically significant for these scales (*p* < 0.0005) (see Table 2 for details). Three of five scales (Physical, Role, and Social Functioning) show a better condition in patients with a neobladder. The Emotional Functioning scale had not a significant group effect, regardless of time points. And the Cognitive Functioning scale did not emphasize time or group effect. Concerning the nine symptoms/items' scales, the score decreased from T0 to T2 for both groups. No interaction was highlighted, too. The effect of time was significant for all the scales (*p* < 0.0005) and the group effect was significant for the following items: nausea (*p* = 0.0027), pain (*p* 0.0005), dyspnea (*p* = 0.012), insomnia (*p* = 0.0004), constipation (*p* = 0.003), indicating a better condition in patients with a neobladder. The Global Health Status scale did not present a significant group effect (*p* = 0.275), but a significant time effect (*p* = 0.0005).

**TABLE 2:** EORTC‐QLQ‐C30. ANOVA mixed model results about matched population.

Scale	Interaction?	Main time effect	Main group effect
Global health status	No	*p* = 0.003	0.326
Physical functioning	No	*p* < 0.0005	0.002
Role functioning	No	*p* < 0.0005	0.001
Emotional functioning	No	*p* < 0.0005	0.051
Cognitive functioning	No	*p* = 0.498	0.07
Social functioning	No	*p* < 0.0005	0.018
Fatigue	No	*p* < 0.0005	0.06
Nausea	No	*p* < 0.0005	0.0027
Pain	No	*p* < 0.0005	0.0005
Dyspnea	No	*p* < 0.0005	0.012
Insomnia	No	*p* < 0.0005	0.004
Appetite	No	*p* < 0.0005	0.237
Constipation	No	*p* < 0.0005	0.003
Diarrhea	No	*p* < 0.0005	0.078
Financial difficulties	No	*p* < 0.0005	0.075

Significant *p* Value in gray.

#### The PGWBI

All six scales (anxiety, depressed mood, positive well‐being, self‐control, general health, vitality) present a significant time effect independently from the group. No significant difference has been highlighted between the groups (Table 3).

**TABLE 3:** The PGWBI. ANOVA mixed model results among the matched population. Significant p Value in gray.

Scale	Interaction?	Main time effect	Main group effect
Anxiety	No	*p* < 0.0005	0.79
Depressed mood	No	*p* < 0.0005	0.861
Positive wellbeing	No	*p* < 0.0005	0.3
Self control	No	*p* < 0.0005	0.85
General health	No	*p* < 0.0005	0.56
Vitality	No	*p* < 0.0005	0.517

Significant *p* Value in gray.

#### The HADS and PSQI

Both the scores for anxiety and depression decreased from *T*_0_ to *T*_2_ (see Table 4), showing an improvement in these two aspects. Results from the ANOVA mixed model highlight that there is a significant effect of time but no significant group effect, as already seen in other tests. This means that scores are significantly changing with time, but the groups' scores are not so far from each other.

**TABLE 4:** The HADS. ANOVA mixed model results among the matched population. Significant p Value in gray.

Scale	Interaction?	Main time effect	Main group effect
Anxiety	No	*p* < 0.0005	0.231
Depression	No	*p* < 0.0005	0.192

Significant *p* Value in gray.

## DISCUSSION

Disease‐free survivors of the three major urological cancers (prostate, kidneys, and bladder) show a similar quality of life to each other and it is comparable to the general population.^20^ We think it is important to find the method to choose the right urinary diversion by entering quality of life and further new biomarkers.^21^ Statistically significant differences can be seen in almost all aspects examined, considering the evolution of the self‐perception of health's state in the postoperative period. Statistically significant differences between the two groups of urinary diversions were seen regarding different functional aspects and symptoms in favor of orthotopic urinary diversion.^22^ We must consider that the choice of urinary diversion is guided mainly by the will of the patient. But the indication has given at last by the surgeon, who must evaluate it both in terms of surgical outcomes and about the patient's postoperative management.^23^ From the population's description, we see a significant difference between the two diversions groups regarding age and comorbidities (expressed by the Charlson Comorbidity Index). Their inhomogeneity may lead to a different perception of physical and psychological health. For example, a 60‐year‐old patient could have different expectations and needs from an older one. Instead, the improvement of the same topics with time could be seen as a new balance state with their diversions, with a new body image, and habits, and feeling confident with possible complications in their management.^24^ The decrease in the personal degree of concern about the disease could be another reason to justify the improvement at *T*_2_ time compared to *T*_0_ (the general rate of relapse was 13.4% and 12‐months‐Clavien‐Dindo at least 3b was 3.8%). A paired comparison with a PSM help us to analyze in a better comparative way baseline characteristics and outcomes. We have to consider that the diversion choice depends on the patient's characteristics, too.^25^ Postsurgical complications had yet shown to worsen QoL, so we explored only two of the many diversions routinely used, because of our broad experience and our low rate of complications, similar for each diversion.^26^ The questionnaires agree about the fact that time is a significant key factor to improve psycho‐physical health. Equivalent items analyzed by different questionnaires show the same results. The orthotopic diversion shows better scores with a greater increase in the QoL over time, although other studies show the same only for specific areas. Faced with the choice among urinary diversions, it remains mandatory to consider the patient's characteristics, comorbidities, expectations, personal needs, and the disease's characteristics. It should be imperative to set up pre and postsurgery training for the patient and the caregiver, also considering the presence of support and counseling groups, as established in other studies that show an improvement in terms of anxiety and depression. We do not find the best urinary diversion ever. Even whit a neobladder the patient could have to deal with incontinence or intermittent catheterization. Additionally, the neobladder is more expensive in terms of care, hospitalization, and surgery, but it shows a better cost‐effectiveness ratio. We need more studies as there are few randomized controlled trials on the subject and even fewer studies that consider patient‐related outcomes as an evaluation criterion. However, it is comforting to know that patients are generally satisfied with their condition over time, whatever urinary diversion has been chosen.

## AUTHOR CONTRIBUTIONS

**Palermo Giuseppe:** Conceptualization; validation. **Bizzarri Francesco Pio:** Methodology; software; data curation; formal analysis; writing – review and editing. **Scarciglia Eros:** Writing – original draft; conceptualization; methodology; data curation. **Sacco Emilio:** Supervision. **Moosavi Seyed Koosha:** Supervision. **Russo Pierluigi:** Supervision. **Gavi Filippo:** Supervision. **Filomena Giovanni Battista:** Supervision. **Rossi Francesco:** Supervision. **Campetella Marco:** Supervision. **Totaro Angelo:** Supervision. **Foschi Nazario:** Supervision. **Racioppi Marco:** Conceptualization; visualization; investigation; supervision.

## CONFLICT OF INTEREST STATEMENT

We have no potencial conflicts of interest.

## APPROVAL OF THE RESEARCH PROTOCOL BY AN INSTITUTIONAL REVIEWER BOARD

The study has been approved by the Ethics Committee (ID 2882, approved by the EC on 05/12/2019).

## INFORMED CONSENT

We have provided patients with informed consent of enrollment approved by our ethics committee.

## REGISTRY AND THE REGISTRATION NO. OF THE STUDY/TRIAL

N/A.

## ANIMAL STUDIES

N/A.
